# Supplementary material for: Epidemiology, literacy, risk factors, and clinical status of oral cancer in East Africa: A scoping review
Source: PLoS One. 2025 Feb 21;20(2):e0317217. doi: 10.1371/journal.pone.0317217 (PMC11844884; doi:10.1371/journal.pone.0317217)
Supplement: S1 Table — (DOCX) [file pone.0317217.s001.docx]

**S1 Table. Search strings used on PubMed database.**

| Tag | Search objectives | Search strings |
| --- | --- | --- |
| #1 | To search for literature on oral cancer | ((((((oral cancer[Title/Abstract]) OR (oral squamous cell carcinoma[Title/Abstract])) OR (oropharyngeal cancer[Title/Abstract])) OR (oral cavity cancer[Title/Abstract])) OR (mouth cancer[Title/Abstract])) OR (cancer of the lip[Title/Abstract])) OR (oral malignant neoplas*[Title/Abstract]) |
| #2 | To search for literature on East African countries | ((((((((((((East Africa[Title/Abstract]) OR (Comoros[Title/Abstract])) OR (Djibouti[Title/Abstract])) OR (Ethiopia[Title/Abstract])) OR (Eritrea[Title/Abstract])) OR (Kenya[Title/Abstract])) OR (Rwanda[Title/Abstract])) OR (Seychelles[Title/Abstract])) OR (Somalia[Title/Abstract])) OR (South Sudan[Title/Abstract])) OR (Sudan[Title/Abstract])) OR (Tanzania[Title/Abstract])) OR (Uganda[Title/Abstract]) |
| #3 | To search for literature on oral cancer in East Africa | (#1) AND (#2) |
