# Supplementary material for: Epidemiology, literacy, risk factors, and clinical status of oral cancer in East Africa: A scoping review
Source: PLoS One. 2025 Feb 21;20(2):e0317217. doi: 10.1371/journal.pone.0317217 (PMC11844884; doi:10.1371/journal.pone.0317217)
Supplement: S2 Table — (DOCX) [file pone.0317217.s002.docx]

**S2 Table. Search strings used on SCOPUS database.**

| Tag | Search objectives | Search strings |
| --- | --- | --- |
| #1 | To search for literature on oral cancer | ( TITLE-ABS-KEY ( "oral cancer" )  OR  TITLE-ABS-KEY ( "oral squamous cell carcinoma" )  OR  TITLE-ABS-KEY ( "oropharyngeal cancer" )  OR  TITLE-ABS-KEY ( "oral cavity cancer" )  OR  TITLE-ABS-KEY ( "mouth cancer" )  OR  TITLE-ABS-KEY ( "cancer of the lip" )  OR  TITLE-ABS-KEY ( "oral malignant neoplas*" )  OR  TITLE-ABS-KEY ( "lip cancer" )  ) |
| #2 | To search for literature on East African countries | ( TITLE-ABS-KEY ( "East Africa" ) OR TITLE-ABS-KEY ( burundi ) OR TITLE-ABS-KEY ( comoros ) OR TITLE-ABS-KEY ( djibouti ) OR TITLE-ABS-KEY ( ethiopia ) OR TITLE-ABS-KEY ( eritrea ) OR TITLE-ABS-KEY ( kenya ) OR TITLE-ABS-KEY ( rwanda ) OR TITLE-ABS-KEY ( seychelles ) OR TITLE-ABS-KEY ( somalia ) OR TITLE-ABS-KEY ( "South Sudan" ) OR TITLE-ABS-KEY ( sudan ) OR TITLE-ABS-KEY ( tanzania ) OR TITLE-ABS-KEY ( uganda ) ) |
| #3 | To search for literature on oral cancer in East Africa | #1 AND #2 |
