# Supplementary material for: Epidemiology, literacy, risk factors, and clinical status of oral cancer in East Africa: A scoping review
Source: PLoS One. 2025 Feb 21;20(2):e0317217. doi: 10.1371/journal.pone.0317217 (PMC11844884; doi:10.1371/journal.pone.0317217)
Supplement: S3 Table — (DOCX) [file pone.0317217.s003.docx]

**S3 Table. Search strings used on AMED – The Allied and Complementary Medicine Database, APA PsycArticles, APA PsycInfo, CINAHL Ultimate, Dentistry & Oral Sciences Source, Psychology and Behavioral Sciences Collection, SPORTDiscus with Full Text databases (via EBSCOHost interface).**

| Tag | Search objectives | Search strings |
| --- | --- | --- |
| S1 | To search for literature on oral cancer | AB Oral cancer OR AB oral squamous cell carcinoma OR AB oropharyngeal cancer OR AB oral cavity cancer OR AB mouth cancer OR AB cancer of the lip OR AB oral malignant neoplas* |
| S2 | To search for literature on East African countries | AB Burundi OR AB Comoros OR AB Djibouti OR AB Ethiopia OR AB Eritrea OR AB Kenya OR AB Rwanda OR AB Seychelles OR AB Somalia OR AB South Sudan OR AB Sudan OR AB Tanzania OR AB Uganda |
| S3 | To search for literature on oral cancer in East Africa | S1 AND S2 |
