# Supplementary material for: Epidemiology, literacy, risk factors, and clinical status of oral cancer in East Africa: A scoping review
Source: PLoS One. 2025 Feb 21;20(2):e0317217. doi: 10.1371/journal.pone.0317217 (PMC11844884; doi:10.1371/journal.pone.0317217)
Supplement: S4 Table — (DOCX) [file pone.0317217.s004.docx]

**S4 Table. List of literature considered for full text screening.**

| **No.** | **Citation** | **Included** | **Excluded (with reasons)** |
| --- | --- | --- | --- |
| 1 | Anass MA, G Ahmed H. A Case-Control Study of Oral Epithelial Proliferative Markers among Sudanese Toombak Dippers Using Micronuclei Assay, Argyrophilic Nucleolar Organizer Region, Papanicolaou and Crystal Violet Methods. Rare Tumors. 2013 Jul 17;5(3):e31. doi: 10.4081/rt.2013.e31. PMID: 24179643; PMCID: PMC3804806. | Yes |  |
| 2 | Cubey RB. A consideration of the aetiology of lip plug carcinoma with special reference to local geographical tumour incidence. Br J Cancer. 1974 Jul;30(1):95-100. doi: 10.1038/bjc.1974.117. PMID: 4411612; PMCID: PMC2009187. |  | Yes (old publication) |
| 3 | Sami A, Elimairi I, Ryan CA, Stanton C, Patangia D, Ross RP. Altered oral microbiome in Sudanese Toombak smokeless tobacco users carries a newly emerging risk of squamous cell carcinoma development and progression. Sci Rep. 2023 Apr 24;13(1):6645. doi: 10.1038/s41598-023-32892-y. PMID: 37095112; PMCID: PMC10125980. | Yes |  |
| 4 | Dimba EA, Gichana J, Limo AK, Wakoli KA, Chindia ML, Awange DO. An audit of oral diseases at a Nairobi centre, 2000-2004. Int Dent J. 2007 Dec;57(6):439-44. doi: 10.1111/j.1875-595x.2007.tb00147.x. PMID: 18265777. | Yes |  |
| 5 | Mohamed N, Litlekalsøy J, Ahmed IA, Martinsen EMH, Furriol J, Javier-Lopez R, Elsheikh M, Gaafar NM, Morgado L, Mundra S, Johannessen AC, Osman TA, Nginamau ES, Suleiman A, Costea DE. Analysis of Salivary Mycobiome in a Cohort of Oral Squamous Cell Carcinoma Patients From Sudan Identifies Higher Salivary Carriage of *Malassezia* as an Independent and Favorable Predictor of Overall Survival. Front Cell Infect Microbiol. 2021 Oct 12;11:673465. doi: 10.3389/fcimb.2021.673465. PMID: 34712619; PMCID: PMC8547610. | Yes |  |
| 6 | Loro LL, Vintermyr OK, Ibrahim SO, Idris AM, Johannessen AC. Apoptosis and expression of Bax and Bcl-2 in snuff- and non-snuff associated oral squamous cell carcinomas. Anticancer Res. 2000 Sep-Oct;20(5A):2855-60. PMID: 11062693. | Yes |  |
| 7 | Boulos PB, El Masri SH. Carcinoma of the oesophagus in the Sudan. Trop Geogr Med. 1977 Jun;29(2):150-4. PMID: 562007. |  | Yes (old publication) |
| 8 | Gaafar NM, Osman TA, Ahmed IA, Elsheikh M, Dongre H, Jacobsen MR, Mohamed NG, Fromreide S, Suleiman AM, Johannessen AC, Nginamau ES, Costea DE. Characterization of immune cell infiltrate in tumor stroma and epithelial compartments in oral squamous cell carcinomas of Sudanese patients. Clin Exp Dent Res. 2022 Feb;8(1):130-140. doi: 10.1002/cre2.501. Epub 2021 Oct 9. PMID: 34626165; PMCID: PMC8874073. | Yes |  |
| 9 | Okumu SB, Chindia ML, Gathece LW, Dimba EA, Odhiambo W. Clinical features and types of paediatric orofacial malignant neoplasms at two hospitals in Nairobi, Kenya. J Craniomaxillofac Surg. 2012 Jan;40(1):e8-14. doi: 10.1016/j.jcms.2011.01.020. Epub 2011 Mar 3. PMID: 21376609. | Yes |  |
| 10 | Eltohami YI, Suleiman AM. Clinical Presentation of Wide Field of Cancerization Associated with Oral Squamous Cell Carcinoma. Int J Dent. 2023 Mar 17;2023:7530295. doi: 10.1155/2023/7530295. PMID: 36969377; PMCID: PMC10038738. | Yes |  |
| 11 | Elimairi I, Altay MA, Abdoun O, Elimairi A, Tozoglu S, Baur DA, Quereshy F. Clinical relevance of the utilization of vital Lugol's iodine staining in detection and diagnosis of oral cancer and dysplasia. Clin Oral Investig. 2017 Mar;21(2):589-595. doi: 10.1007/s00784-016-1925-x. Epub 2016 Aug 5. PMID: 27491775. | Yes |  |
| 12 | Idris AM, Ahmed HM, Mukhtar BI, Gadir AF, el-Beshir EI. Descriptive epidemiology of oral neoplasms in Sudan 1970-1985 and the role of toombak. Int J Cancer. 1995 Apr 10;61(2):155-8. doi: 10.1002/ijc.2910610202. PMID: 7705940. |  | Yes (old publication) |
| 13 | Jalouli MM, Jalouli J, Sapkota D, Ibrahim SO, Sand L, Hirsch JM. Differential expression of apoptosis, cell cycle regulation and intermediate filament genes in oral squamous cell carcinomas associated with toombak use in Sudan. Anticancer Res. 2011 Oct;31(10):3345-51. PMID: 21965745. | Yes |  |
| 14 | Gaafar NM, Osman TA, Elsheikh M, Ahmed IA, Dongre H, Fromreide S, Suleiman AM, Johannessen AC, Nginamau ES, Costea DE. Epithelial PD-L1 expression at tumor front predicts overall survival in a cohort of oral squamous cell carcinomas from Sudan. Clin Exp Dent Res. 2022 Dec;8(6):1467-1477. doi: 10.1002/cre2.666. Epub 2022 Sep 30. PMID: 36177667; PMCID: PMC9760153. | Yes |  |
| 15 | Ibrahim SO, Warnakulasuriya KA, Idris AM, Hirsch JM, Johnson NW, Johannessen AC. Expression of keratin 13, 14 and 19 in oral hyperplastic and dysplastic lesions from Sudanese and Swedish snuff-dippers: association with human papillomavirus infection. Anticancer Res. 1998 Jan-Feb;18(1B):635-45. PMID: 9584046. |  | Yes (old publication) |
| 16 | Ibrahim SO, Bertelsen B, Kalvenes MB, Idris AM, Vasstrand EN, Nilsen R, Johannessen AC. Expression of keratin 13, 14 and 19 in oral squamous cell carcinomas from Sudanese snuff dippers: lack of association with human papillomavirus infection. APMIS. 1998 Oct;106(10):959-69. doi: 10.1111/j.1699-0463.1998.tb00246.x. PMID: 9833698. |  | Yes (old publication) |
| 17 | Ndayisabye H, Ndagijimana A, Biracyaza E, Umubyeyi A. Factors Associated With Oral Cancer Adverse Outcome at the Rwanda Military Hospital, a Retrospective Cross-Sectional Study. Front Oral Health. 2022 Mar 18;3:844254. doi: 10.3389/froh.2022.844254. PMID: 35368313; PMCID: PMC8971924. | Yes |  |
| 18 | Mohamed N, van de Goor R, El-Sheikh M, Elrayah O, Osman T, Nginamau ES, Johannessen AC, Suleiman A, Costea DE, Kross KW. Feasibility of a Portable Electronic Nose for Detection of Oral Squamous Cell Carcinoma in Sudan. Healthcare (Basel). 2021 May 3;9(5):534. doi: 10.3390/healthcare9050534. PMID: 34063592; PMCID: PMC8147635. | Yes |  |
| 19 | Ibrahim SO, Aarsaether N, Holsve MK, Kross KW, Heimdal JH, Aarstad JH, Liavaag PG, Elgindi OA, Johannessen AC, Lillehaug JR, Vasstrand EN. Gene expression profile in oral squamous cell carcinomas and matching normal oral mucosal tissues from black Africans and white Caucasians: the case of the Sudan vs. Norway. Oral Oncol. 2003 Jan;39(1):37-48. doi: 10.1016/s1368-8375(02)00018-0. PMID: 12457720. | Yes |  |
| 20 | Ibrahim SO, Johannessen AC, Idris AM, Hirsch JM, Vasstrand EN, Magnusson B, Nilsen R. Immunohistochemical detection of p53 in non-malignant and malignant oral lesions associated with snuff dipping in the Sudan and Sweden. Int J Cancer. 1996 Dec 11;68(6):749-53. doi: 10.1002/(SICI)1097-0215(19961211)68:6<749::AID-IJC10>3.0.CO;2-W. PMID: 8980178. |  | Yes (old publication) |
| 21 | Ginawi IA, Mahgoub EA, Ahmed HG. Immunophenotyping of HPV Types 16 and 18 among Sudanese Patients with Oral Lesions. Oman Med J. 2012 May;27(3):201-6. doi: 10.5001/omj.2012.45. PMID: 22811767; PMCID: PMC3394353. | Yes |  |
| 22 | Ahmed HG, Mahgoob RM. Impact of Toombak dipping in the etiology of oral cancer: gender-exclusive hazard in the Sudan. J Cancer Res Ther. 2007 Apr-Jun;3(2):127-30. doi: 10.4103/0973-1482.34696. PMID: 17998740. | Yes |  |
| 23 | Ibrahim SO, Lillehaug JR, Dolphine O, Johnson NW, Warnakulasuriya KA, Vasstrand EN. Mutations of the cell cycle arrest gene p21WAF1, but not the metastasis-inducing gene S100A4, are frequent in oral squamous cell carcinomas from Sudanese toombak dippers and non-snuff-dippers from the Sudan, Scandinavia, USA and UK. Anticancer Res. 2002 May-Jun;22(3):1445-51. PMID: 12168821. | Yes |  |
| 24 | Ibrahim SO, Vasstrand EN, Johannessen AC, Idris AM, Magnusson B, Nilsen R, Lillehaug JR. Mutations of the p53 gene in oral squamous-cell carcinomas from Sudanese dippers of nitrosamine-rich toombak and non-snuff-dippers from the Sudan and Scandinavia. Int J Cancer. 1999 May 17;81(4):527-34. doi: 10.1002/(sici)1097-0215(19990517)81:4<527::aid-ijc4>3.0.co;2-2. PMID: 10225439. |  | Yes (old publication) |
| 25 | Onyango JF, Omondi BI, Njiru A, Awange OO. Oral cancer at Kenyatta National Hospital, Nairobi. East Afr Med J. 2004 Jun;81(6):318-21. doi: 10.4314/eamj.v81i6.9182. PMID: 16167680. | Yes |  |
| 26 | Babiker TM, Osman KA, Mohamed SA, Mohamed MA, Almahdi HM. Oral Cancer Awareness Among Dental Patients in Omdurman, Sudan: a cross-sectional Study. BMC Oral Health. 2017 Mar 23;17(1):69. doi: 10.1186/s12903-017-0351-z. PMID: 28335762; PMCID: PMC5364606. | Yes |  |
| 27 | Ahmed NHM, Naidoo S. Oral Cancer Knowledge, Attitudes, and Practices among Dentists in Khartoum State, Sudan. J Cancer Educ. 2019 Apr;34(2):291-296. doi: 10.1007/s13187-017-1300-x. PMID: 29151257. | Yes |  |
| 28 | Cameron HM. Oral tumours in Kenya. Pathobiology. 1973 Oct 3;39(3-4):187-95. |  | Yes (old publication) |
| 29 | Sand L, Jalouli MM, Jalouli J, Sapkota D, Ibrahim SO. p53 Codon 72 polymorphism in oral exfoliated cells in a Sudanese population. In Vivo. 2012 Jan-Feb;26(1):59-62. PMID: 22210716. | Yes |  |
| 30 | Butt FM, Chindia ML, Rana F, Machigo FG. Pattern of head and neck malignant neoplasms in HIV-infected patients in Kenya. Int J Oral Maxillofac Surg. 2008 Oct;37(10):907-11. doi: 10.1016/j.ijom.2008.07.019. Epub 2008 Sep 9. PMID: 18783921. | Yes |  |
| 31 | Osman TA, Satti AA, Boe OE, Yang YH, Ibrahim SO, Suleiman AM. Pattern of malignant tumors registered at a referral oral and maxillofacial hospital in Sudan during 2006 and 2007. J Cancer Res Ther. 2010 Oct-Dec;6(4):473-7. doi: 10.4103/0973-1482.77112. PMID: 21358083. | Yes |  |
| 32 | Nabirye RC, Kamulegeya A. Public knowledge about oral cancer in Uganda: a free dental camp experience. J Health Res. 2019;33(4):270-279. doi: 10.1108/jhr-07-2018-0062. Epub 2019 Jul 7. PMID: 32051930; PMCID: PMC7015528. | Yes |  |
| 33 | Al-Hakimi HA, Othman AE, Mohamed OG, Saied AM, Ahmed WA. Public Knowledge of Oral Cancer and Modelling of Demographic Background Factors Affecting this Knowledge in Khartoum State, Sudan. Sultan Qaboos Univ Med J. 2016 Aug;16(3):e335-40. doi: 10.18295/squmj.2016.16.03.012. Epub 2016 Aug 19. PMID: 27606114; PMCID: PMC4996297. | Yes |  |
| 34 | Eltohami YI, Sulaiman AM. Recurrence in Oral Squamous Cell Carcinoma Associated with Wide Field of Cancerization: Analysis of 93 Cases. Indian J Otolaryngol Head Neck Surg. 2023 Sep;75(3):1329-1335. doi: 10.1007/s12070-023-03548-0. Epub 2023 Feb 13. PMID: 37636723; PMCID: PMC10447784. | Yes |  |
| 35 | Babiker AY, Eltom FM, Abdalaziz MS, Rahmani A, Abusail S, Ahmed HG. Screening for high risk human papilloma virus (HR-HPV) subtypes, among Sudanese patients with oral lesions. Int J Clin Exp Med. 2013 Apr 12;6(4):275-81. PMID: 23641304; PMCID: PMC3631552. | Yes |  |
| 36 | Elbeshir EI, Abeen HA, Idris AM, Abbas K. Snuff dipping and oral cancer in Sudan: a retrospective study. Br J Oral Maxillofac Surg. 1989 Jun;27(3):243-8. doi: 10.1016/0266-4356(89)90152-6. PMID: 2742810. |  | Yes (Old publication) |
| 37 | Ahmed HG, Idris AM, Ibrahim SO. Study of oral epithelial atypia among Sudanese tobacco users by exfoliative cytology. Anticancer Res. 2003 Mar-Apr;23(2C):1943-9. PMID: 12820484. | Yes |  |
| 38 | Elasbali AM, Ahmed EAEA, Ahmed HG. Study on immunohistochemical expression of P53 among Sudanese tobacco users with oral squamous cell carcinoma. Int J Integr Biol. 2013;14(2):74-77. |  | Yes (Inaccessible full text) |
| 39 | Asio J, Kamulegeya A, Banura C. Survival and associated factors among patients with oral squamous cell carcinoma (OSCC) in Mulago hospital, Kampala, Uganda. Cancers Head Neck. 2018 Oct 26;3:9. doi: 10.1186/s41199-018-0036-6. PMID: 31093362; PMCID: PMC6460549. | Yes |  |
| 40 | Idris AM, Prokopczyk B, Hoffmann D. Toombak: a major risk factor for cancer of the oral cavity in Sudan. Prev Med. 1994 Nov;23(6):832-9. doi: 10.1006/pmed.1994.1141. PMID: 7855117. |  | Yes (Old publication) |
| 41 | Idris AM, Ahmed HM, Malik MO. Toombak dipping and cancer of the oral cavity in the Sudan: a case-control study. Int J Cancer. 1995 Nov 15;63(4):477-80. doi: 10.1002/ijc.2910630402. PMID: 7591252. |  | Yes (Old publication) |
| 42 | Idris AM, Warnakulasuriya KA, Ibrahim YE, Nielsen R, Cooper D, Johnson NW. Toombak-associated oral mucosal lesions in Sudanese show a low prevalence of epithelial dysplasia. J Oral Pathol Med. 1996 May;25(5):239-44. doi: 10.1111/j.1600-0714.1996.tb01378.x. PMID: 8835821. |  | Yes (Old publication) |
| 43 | Sheikh ME, Suleiman A, Satti A, O'Sullivan EM. Translation, linguistic validation and reliability of FACT-H&N questionnaire in Oral Cancer patients in Sudan. J Patient Rep Outcomes. 2022 Sep 16;6(1):98. doi: 10.1186/s41687-022-00507-1. PMID: 36114352; PMCID: PMC9481800. |  | Yes (Wrong study objective) |
| 44 | Ibrahim SO, Miron T, Krohn M, Amaratunga AN, Warnakulasauriya S, Vasstrand EN. Tumour-associated Proteins in Oral Squamous Cell Carcinomas by Proteomics. Cancer Genomics Proteomics. 2005 Nov-Dec;2(6):353-363. Epub 2005 Nov 1. PMID: 31394652. | Yes |  |
