# Supplementary material for: Epidemiology, literacy, risk factors, and clinical status of oral cancer in East Africa: A scoping review
Source: PLoS One. 2025 Feb 21;20(2):e0317217. doi: 10.1371/journal.pone.0317217 (PMC11844884; doi:10.1371/journal.pone.0317217)
Supplement: S6 Table — (DOCX) [file pone.0317217.s006.docx]

**S6 Table. Quality appraisal outcomes of the appraised quantitative non-randomized studies using the Mixed Methods Appraisal Tool**

| **No.** | **Author (Year)** | **Study Design** | **Responses to the Appraisal Questions for Quantitative Non-randomized Studies** | | | | | | | **Scored Points (out of a Total of 7 Points)** | **Grade** |
| --- | --- | --- | --- | --- | --- | --- | --- | --- | --- | --- | --- |
|  |  |  | Are there clear research questions? | Do the collected data allow to address the research questions? | Are the participants representative of the largest population? | Are measurements appropriate regarding both the outcome and intervention (or exposure)? | Are there complete outcome data? | Are the confounders accounted for in the design and analysis? | During the study period, is the intervention administered (or exposure occurred) as intended? |  |  |
| 1 | Anass & Ahmed (2013) | Case-control study | I can’t tell | I can’t tell | Yes | Yes | Yes | No | Yes | 5 | Above average quality |
| 2 | Sami et al., (2023) | Case-control study | I can’t tell | I can’t tell | Yes | Yes | Yes | No | Yes | 5 | Above average quality |
| 3 | Dimba et al., (2007) | Cohort study (retrospective) | I can’t tell | I can’t tell | Yes | No | No | No | I can’t tell | 2.5 | Below average quality |
| 4 | Mohamed et al., (2021) | Cohort study (prospective) | I can’t tell | I can’t tell | Yes | Yes | Yes | Yes | Yes | 6 | Above average quality |
| 5 | Loro et al., (2000) | Cross-sectional analytical study | I can’t tell | I can’t tell | Yes | Yes | Yes | No | I can’t tell | 4.5 | Above average quality |
| 6 | Gaafar et al., (2022) | Cohort study (prospective) | I can’t tell | I can’t tell | I can’t tell | Yes | Yes | Yes | Yes | 5.5 | Above average quality |
| 7 | Okumu et al., (2012) | Cross-sectional analytical study | I can’t tell | I can’t tell | Yes | I can’t tell | Yes | No | No | 3.5 | Average quality |
| 8 | Eltohami & Suleiman, (2023) | Cohort study (prospective) | I can’t tell | I can’t tell | Yes | Yes | Yes | No | Yes | 5.0 | Above average quality |
| 9 | Elimairi et al., (2017) | Cross-sectional analytical study | I can’t tell | I can’t tell | Yes | Yes | Yes | No | Yes | 5.0 | Above average quality |
| 10 | Jalouli et al., (2011) | Case-control study | I can’t tell | I can’t tell | Yes | Yes | Yes | No | Yes | 5.0 | Above average quality |
| 11 | Gaafar, Osman, Elsheikh, et al. (2022) | Cohort study (prospective) | 0.5 | I can’t tell | No | I can’t tell | Yes | No | Yes | 3.5 | Average quality |
| 12 | Ndayisabye et al., (2022) | Cross-sectional analytical study | I can’t tell | I can’t tell | No | Yes | No | No | Yes | 3.0 | Below average |
| 13 | Mohamed, Van De Goor, et al., (2021) | Cohort study (prospective) | I can’t tell | I can’t tell | I can’t tell | Yes | Yes | 0 | Yes | 4.5 | Above average quality |
| 14 | Ibrahim et al., (2003) | Cohort study (prospective) | No | No | I can’t tell | Yes | Yes | No | Yes | 3.5 | Average quality |
| 15 | Ginawi et al., (2012) | Cohort study (retrospective) | I can’t tell | I can’t tell | No | Yes | Yes | No | Yes | 4.0 | Above average quality |
| 16 | Ibrahim et al., (2002) | Cross-sectional analytical study | I can’t tell | I can’t tell | I can’t tell | Yes | I can’t tell | No | I can’t tell | 3.5 | Average quality |
| 17 | Babiker et al., (2017) | Cross-sectional analytical study | I can’t tell | I can’t tell | No | I can’t tell | Yes | No | Yes | 3.5 | Average quality |
| 18 | Ahmed & Naidoo, (2019) | Cross-sectional analytical study | I can’t tell | I can’t tell | I can’t say | Yes | Yes | No | I can’t say | 4.0 | Above quality |
| 19 | Sand et al., (2012) | Cohort study (prospective) | I can’t tell | I can’t tell | I can’t say | Yes | Yes | No | Yes | 4.5 | Above average quality |
| 20 | Al-Hakimi et al., (2016) | Cross-sectional analytical study | I can’t tell | I can’t tell | No | Yes | Yes | No | Yes | 4.0 | Above average quality |
| 21 | Eltohami & Sulaiman, (2023) | Cohort study (prospective) | Yes | I can’t tell | I can’t tell | I can’t tell | I can’t tell | No | No | 3.0 | Below average |
| 22 | Asio et al., (2018) | Cohort study (retrospective) | Yes | Yes | Yes | Yes | No | No | No | 4.0 | Above average |
| Yes – 1 point; I can’t tell – 0.5 point; No – 0 point; Below average – <3.5/7 points; Average – 3.5/7 points; Above average – >3.5/7 points and above | | | | | | | | | | | |
