# Supplementary material for: Epidemiology, literacy, risk factors, and clinical status of oral cancer in East Africa: A scoping review
Source: PLoS One. 2025 Feb 21;20(2):e0317217. doi: 10.1371/journal.pone.0317217 (PMC11844884; doi:10.1371/journal.pone.0317217)
Supplement: S7 Table — (DOCX) [file pone.0317217.s007.docx]

**S7 Table. Quality appraisal outcomes of the appraised quantitative descriptive study using the Mixed Methods Appraisal**

| **No.** | **Author (Year)** | **Study Design** | **Responses to the Appraisal Questions for Quantitative Descriptive Studies** | | | | | | | **Scored Points (out of a Total of 7 Points)** | **Grade** |
| --- | --- | --- | --- | --- | --- | --- | --- | --- | --- | --- | --- |
|  |  |  | Are there clear research questions? | Do the collected data allow to address the research questions? | Is the sampling strategy relevant to address the research question? | Is the sample representative of the target population? | Are the measurements appropriate? | Is the risk of nonresponse bias low? | Is the statistical analysis appropriate to answer the research question? |  |  |
| 1 | Ahmed & Mahgoob, (2007) | Cross-sectional descriptive study | I can’t tell | I can’t tell | No | No | No | I can’t tell | I can’t tell | 2 | Below average |
| 2 | Onyango et al., (2004) | Cohort study (retrospective) | Yes | I can’t tell | Yes | Yes | I can’t tell | I can’t tell | No | 4.5 | Above average |
| 3 | Butt et al., (2008) | Cross sectional descriptive study | No | No | I can’t tell | I can’t tell | I can’t tell | I can’t tell | No | 2 | Below Average |
| 4 | Osman et al., (2010) | Cross sectional descriptive study | I can’t tell | I can’t tell | No | I can’t tell | Yes | No | I can’t tell | 3.0 | Below average |
| 5 | Nabirye & Kamulegeya, (2019) | Cross sectional descriptive study | I can’t tell | I can’t tell | No | No | Yes | No | I can’t tell | 2.5 | Below average |
| 6 | Babiker et al., (2013) | Cohort study (retrospective) | I can’t tell | I can’t tell | No | I can’t tell | Yes | I can’t tell | I can’t tell | 3.5 | Average |
| 7 | Ahmed et al., (2003) | Cohort study (retrospective) | I can’t tell | I can’t tell | Yes | I can’t tell | Yes | I can’t tell | Yes | 5.0 | Above average |
| 8 | Ibrahim et al., (2005) | Cohort study (prospective) | I can’t tell | I can’t tell | Yes | I can’t tell | Yes | No | Yes | 4.5 | Above average |
| Yes – 1 point; I can’t tell – 0.5 point; No – 0 point; Below average – <3.5/7 points; Average – 3.5/7 points; Above average – >3.5/7 points and above | | | | | | | | | | | |
