# Supplementary material for: Epidemiology, literacy, risk factors, and clinical status of oral cancer in East Africa: A scoping review
Source: PLoS One. 2025 Feb 21;20(2):e0317217. doi: 10.1371/journal.pone.0317217 (PMC11844884; doi:10.1371/journal.pone.0317217)
Supplement: S8 Table — (DOCX) [file pone.0317217.s008.docx]

**S8 Table. Quality appraisal outcomes of the appraised qualitative study using the Mixed Methods Appraisal Tool**

| **No.** | **Author (Year)** | **Study Design** | **Responses to the Appraisal Questions for Qualitative Studies** | | | | | | | **Scored Points (out of a Total of 7 Points)** | **Grade** |
| --- | --- | --- | --- | --- | --- | --- | --- | --- | --- | --- | --- |
|  |  |  | Are there clear research questions? | Do the collected data allow to address the research questions? | Is the qualitative approach appropriate to answer the research question? | Are the qualitative data collection methods adequate to address the research question? | Are the findings adequately derived from the data? | Is the interpretation of results sufficiently substantiated by data? | Is there coherence between qualitative data sources, collection, analysis and interpretation? |  |  |
| 1 | Nil | Nil | Nil | Nil | Nil | Nil | Nil | Nil | Nil | Nil | Nil |
| Yes – 1 point; I can’t tell – 0.5 point; No – 0 point; Below average – <3.5/7 points; Average – 3.5/7 points; Above average – >3.5/7 points and above | | | | | | | | | | | |
